# Distribution Analysis of Salvianolic Acids in Myocardial Ischemic Pig Tissues by Automated Liquid Extraction Surface Analysis Coupled with Tandem Mass Spectrometry

**DOI:** 10.1155/2020/8476794

**Published:** 2020-09-14

**Authors:** Qi Qiu, Jinglin Cao, Yu Mu, Yang Lin, Yunnan Zhang, Jing Li, Xiujin Shi

**Affiliations:** ^1^Department of Pharmacy, Beijing Anzhen Hospital, Capital Medical University, Beijing 100029, China; ^2^School of Pharmaceutical Sciences, Capital Medical University, Beijing 100069, China

## Abstract

The distribution of active compounds of traditional Chinese medicine *Salvia miltiorrhiza* Bunge (Chinese name: Danshen) in vivo was determined by establishing a liquid extraction surface analysis coupled with the tandem mass spectrometry (LESA-MS/MS) method. Stability analysis and distribution analysis were designed in the present study using normal animals or a myocardial ischemia model. The model assessment was performed four weeks after surgery, and then three groups were created: a normal-dose group, a model-blank group, and a model-dose group. Meanwhile, Danshen decoction administration began in dose groups and lasted for four weeks. In stability analysis, four salvianolic acids—Danshensu (DSS), caffeic acid (CAA), rosmarinic acid (RA), and salvianolic acid A (SAA)—in kidney tissues from the normal-dose group were detected by LESA-MS/MS under four conditions, and then distribution analysis was conducted in different tissues using the same method. Ejection fraction (EF) and fractional shortening (FS) in animals from two model groups decreased significantly four weeks after surgery (*P* < 0.01) and were improved after four weeks of Danshen decoction administration (*P* < 0.01). Results of stability analysis demonstrated that this method was basically stable since there were no significant differences in signal intensities of DSS, CAA, and SAA under four conditions (*P* > 0.05). Distribution analysis showed the signal intensities of DSS in the liver and kidney and SAA in the heart were higher in the model-dose group than in the normal-dose group (*P* < 0.05 or *P* < 0.01). Signal intensities of RA in the liver and kidney, and SAA in the liver were lower in the model-dose group compared with the normal-dose group (*P* < 0.05 or *P* < 0.01). In conclusion, Danshen decoction has the effect of improving the ischemic condition in a chronic myocardial ischemia model, and the content of two active compounds increased in the targets. These findings contribute to an understanding of the therapeutic role of Danshen in cardiovascular disease.

## 1. Introduction

Cardiovascular disease (CVD), which is defined as a set of diseases and conditions including coronary heart disease (CHD), cerebrovascular disease, and heart failure, has been the leading cause of mortality across the world [1]. It is estimated that by 2030, 23.6 million people will die each year from CVD. CHD, as the most important disease of CVD, has climbed from the seventh leading cause of death in China in 1990 to the second today [2]. Because of the increasing cost of hospitalization for CHD, it has become one of the largest disease burdens in China.

Traditional Chinese medicine (TCM) is a valuable asset for preventing and treating disease. In China, TCM as a complementary therapy has been widely used for CVD. Recent reviews have also suggested that TCM may be beneficial to patients with CVD. The TCM-Danshen is the dry root and rhizome of *Salvia miltiorrhiza* Bunge and was first recorded in Shennong Herbal Classic. It is commonly used in the treatment of cardiovascular system, digestive system, and nervous system diseases. The theory of TCM considers Danshen an important medicine for expanding blood vessels, promoting blood circulation, eliminating blood stasis, and relieving pain [3]. The main chemical constituents of Danshen are divided into water- and liposoluble components [3]. Its water-soluble phenolic acids are salvianolic acids, including Danshensu (DSS), caffeic acid (CAA), rosmarinic acid (RA), and salvianolic acid A (SAA) [4, 5]. These salvianolic acids have pharmacological activities and play a major role in the treatment of CHD [6].

Mapping and quantifying the distribution of drugs in vivo are critical to elucidating their mechanisms of action. The distributions of drugs and their quantities at target sites are closely related to their efficacy and safety. However, analysis of TCMs and their metabolites remains challenging because of the diversity of their compositions, the complexity of biological matrices, and the presence of trace amounts of components and metabolites [7].

In the present study, the distribution of four salvianolic acids (DSS, CAA, RA, and SAA) of Danshen in myocardial ischemic pig tissues was determined using a liquid extraction surface analysis coupled with tandem mass spectrometry (LESA-MS/MS) method which is a fully automated, chip-based method with the characteristics of simplicity and efficiency. An attempt was made to explore the relative amounts and spatial distributions of the target compounds in vivo and the therapeutic effect of Danshen on CHD.

## 2. Materials and Methods

### 2.1. Chemicals and Reagents

**Figure 1:**
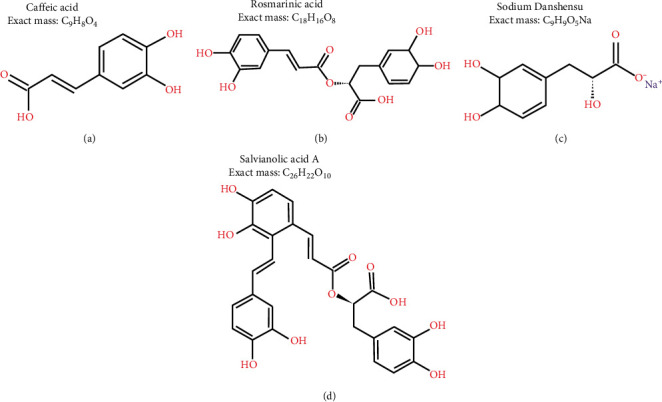
Chemical structures of the compounds investigated in this study.

Standards of CAA (lot no. LL90Q26, 99% purity), DSS sodium salt (lot no. LIA0Q 80, 99% purity), RA (lot no. L970N70, 99% purity), and SAA (lot no. L4B0P55, 99% purity) were purchased from J&K Scientific Ltd (Beijing, China) (Figure 1).

Methanol (CAS no.67-56-1, batch no. 150162, Fisher Scientific UK, Loughborough, UK), ammonium hydroxide (Beijing Chemical Plant, Beijing, China), and high-performance liquid chromatography (HPLC) grade water (CAS no. 7732-18-5, batch no. F8CJ21, DUKSAN, Ansan-si, Korea) were used.

### 2.2. Drug Preparation

All crude drugs were purchased from Beijing Tongrentang Pharmaceutical Co., Ltd. (Beijing, China), including *Salvia miltiorrhiza* Bunge pieces (lot no. SAA291), *Amomum villosum* Lour pieces (lot no. SA1271), and *Santalum album* L. pieces (lot no. SAA271), and identified at Beijing University of Chinese Medicine. Danshen decoction was prepared as follows: 50 g *Salvia miltiorrhiza* Bunge, 7.5 g *Amomum villosum* Lour, and 7.5 g *Santalum album* L. were decocted with water twice and then were extracted by 95% ethanol. After filtering, recovering ethanol, and decompression drying, the major ingredients of Danshen decoction were obtained. Then, the excipient dextrin was added to form the final product. Analysis of high-performance liquid chromatography-mass spectrometry (HPLC-MS) showed the contents of CAA, RA, SAA, and DSS in the Danshen decoction were 0.08 *μ*g/mg, 2.84 *μ*g/mg, 14.84 *μ*g/mg, and 24.54 *μ*g/mg, respectively (see Figures S1–S5 in the Supplementary Material).

### 2.3. Animals

15 male Bama miniature pigs (25 ± 2 kg, 6–10 months, lot no. SCXK2015-0002) were purchased from Tianjin Bainong Laboratory Animal Breeding Technology Co., Ltd. (Tianjin, China). Pigs were housed under standard laboratory conditions, fed twice a day, and given tap water ad libitum. All the animal care and experimental procedures were performed in accordance with the China Physiological Society's “Guiding Principles in the Care and Use of Animals” with the approval from the Animal Care Committee of Beijing Anzhen Hospital, Capital Medical University (no. 0000353).

### 2.4. Surgical Protocol and Groups

**Figure 2:**
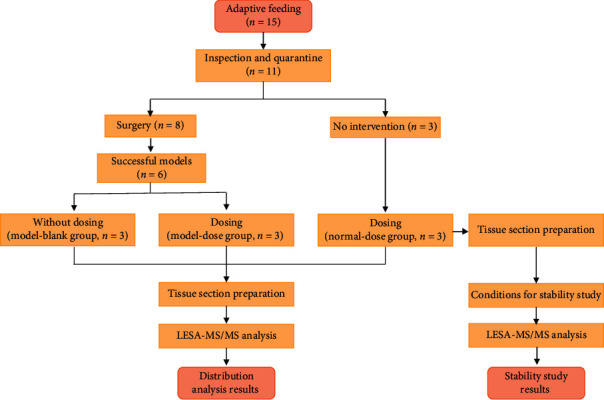
Study design and procedures.

After one week of adaptive feeding, inspection, and quarantine, eleven animals that met test standards were retained. Eight animals were randomly selected to a model building which underwent thoracotomy with an Ameroid constrictor (ø 2.75 mm, Research Instrument SW, USA) placing on the proximal left circumflex artery [8]. Model assessment via coronary angiography (OEC 9900 Elite) and echocardiography (Philips IE33) was performed four weeks after surgery. Six animals which were successfully modelled were randomly divided into a model-blank group (*n* = 3) and a model-dose group (*n* = 3). The other three animals without undergoing surgery were assigned to the normal-dose group. Then Danshen decoction administration was begun in the model-dose group and normal-dose group with the dose of 0.33 g/kg and lasted for four weeks. This choice of dosage was based on a clinical daily dosage of 20 g/60 kg (Figure 2).

### 2.5. Evaluation of Danshen Decoction Effect on Heart Function

Echocardiography was assessed four weeks after surgery and four weeks after administration for each animal, and ejection fraction (EF) and fractional shortening (FS) were obtained by the software.

### 2.6. Tissue Section Preparation

After four weeks of administration, tissues including the kidney, spleen, lung, heart, and liver were harvested from each animal. After washing with PBS, tissues were cut into suitable pieces: kidney (10 × 10 × 8 mm), spleen (10 × 10 × 5 mm), lung (10 × 10 × 5 mm), heart (10 × 10 × 8 mm), and liver (10 × 10 × 8 mm) according to their physiological characteristics, and prepared to be sliced. After embedding the tissues in optimal cutting temperature (OCT) compounds, they were sliced to a thickness of 6 *μ*m at −20°C using a cryomicrotome (CM3600, Leica Microsystems GmbH). Five sections of each tissue were sliced for distribution analysis. Five more sections of each kidney tissue from the normal-dose group were sliced for the stability study. All tissue sections were kept frozen at −80°C until analysed.

### 2.7. Stability Study

#### 2.7.1. Conditions for Stability Study

12 kidney tissue sections were randomly selected from the −80°C freezer after being frozen for 12 h and then were preprocessed in four conditions as described below. Each condition used three sections to detect.

For normal experimental conditions, three tissue sections were rewarmed at room temperature for 30 min. For a repeated rewarming stability investigation, three tissue sections were rewarmed at room temperature for 30 min, then refrozen at −80°C for 12 h, and rewarmed again. This procedure was repeated three times. The third three tissue sections were used in a long-term frozen stability investigation. Sections were stored in a −80°C freezer for 14 days and rewarmed at room temperature for 30 min. The final three tissue sections were used in a stability study of short-term placement at room temperature for 24 h.

#### 2.7.2. LESA-MS/MS Analysis

Tissue sections were analysed on a TriVersa NanoMate (Advion Inc., Ithaca, NY, USA) with a LESA instrument coupled to a 5500 QTRAP MS (AB Sciex, Concord, Ontario). Three points on the preprocessed tissue section were selected for LESA-MS/MS analysis, and the average signal intensity of these points was taken as the detection result. Tissue sections were fixed on the LESA universal adapter plate and scanned by an Epson Perfection V370 scanner. The pictures were processed further by LESA Points software to generate sampling locations and automatic injection. A conductive pipette tip was picked up by the robotic arm of the TriVersa NanoMate to aspirate 1.7 *μ*L of solvent (80/19.9/0.1 v/v/v, methanol : water : ammonium hydroxide). For salvianolic acids, methanol/formic acid and methanol/ammonium hydroxide combinations with water were investigated. An extraction solution of 80/19.9/0.1 methanol/water/ammonium hydroxide (v/v/v) gave the highest analyte response. Subsequently, the tip was placed over specific sampling locations and 0.7 *μ*L of solvent was dispensed onto the surface of the tissue. The liquid junction between the tissue and the pipette tip was maintained for 2 s for analyte extraction, and then the liquid was aspirated back into the tip. The dispensing/aspirating cycle was repeated two more times. After extraction, the analyte was infused into the MS through a multichannel nanoelectrospray ionization (ESI) chip (Advion Inc., Ithaca, NY, USA). A new pipette tip and chip nozzle were used for every sampling location to eliminate cross-contamination.

ESI flow rate was estimated to be 400–500 nL/min. A spray voltage of 1.7 kV and a gas pressure of 0.7 psi were applied in all experiments. Multiple reaction monitoring (MRM) in negative ion mode was used for transitions at the following *m*/*z* values: DSS 196.8 ⟶ 135.0, CAA 179.0 ⟶ 107.0, RA 359.1 ⟶ 161.0, and SAA 493.2 ⟶ 295.0 [9, 10]. The MS parameters were as follows: curtain gas pressure, 10 psi; collision gas pressure, medium; delustering potential, 120 V; entrance potential, 10 V; collision energy, 25 eV; collision cell exit potential, 16 V; and dwell time, 50 ms.

### 2.8. Distribution Analysis

Distribution analysis was performed in the kidney, spleen, lung, heart (ischemic marginal zone of the myocardium), and liver. Three sections of each tissue from each animal were randomly selected for the detection process. The detection process was consistent with the stability study. The experiment was repeated in three animals in each group, and the average signal intensity was taken as the final detection result.

### 2.9. Statistical Analysis

Data were collected using Analyst 1.6.2 software (AB Sciex), and statistical analysis was performed with SPSS version 20.0. All data are presented as the mean ± standard deviation. Statistical analysis was carried out on three or more groups using one-way analysis of variance and Dunnett's test. Statistical analysis of data from repeats was performed by repeated-measures analysis. Values of *P* < 0.05 were considered statistically significant.

## 3. Results

### 3.1. Model Assessment

Four weeks after surgery, coronary angiography showed that the rate of coronary artery stenosis in eight miniature pigs was 100% (see Figure S6 in the Supplementary Material). Echocardiography showed that the EF of six animals was less than 60% (see Figure S7 in the Supplementary Material), which supported the diagnosis of myocardial ischemia.

### 3.2. Effects of Danshen Decoction on Heart Function

**Figure 3:**
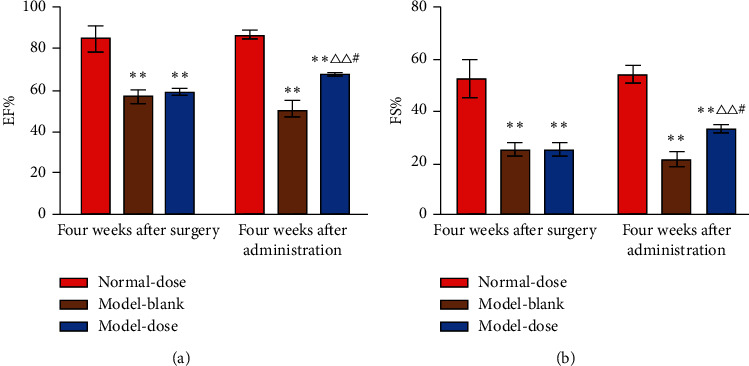
Echocardiography results at different time points between groups. (a) Ejection fractions at different time points between groups. (b) Fractional shortening at different time points between groups. EF: ejection fractions; FS: fractional shortening; Normal-dose: normal-dose group; Model-blank: model-blank group; Model-dose: model-dose group. ^*∗∗*^*P* < 0.01 vs. normal-dose group. Δ^Δ^*P* < 0.01 vs. model-blank group. #*P* < 0.05 vs. model-dose group at four weeks after surgery.

Four weeks after surgery, the EF and FS of pigs in the model-blank group and the model-dose group were significantly lower than those in the normal-dose group (*P* < 0.01). There was no significant difference in the EF and FS between the two model groups (*P* > 0.05). After treated with Danshen decoction for four weeks, EF and FS of pigs in the model-dose group were improved significantly than before (*P* < 0.05) and had obvious differences when compared with the model-blank group (*P* < 0.01) (Figure 3).

### 3.3. Stability Study Results

**Figure 4:**
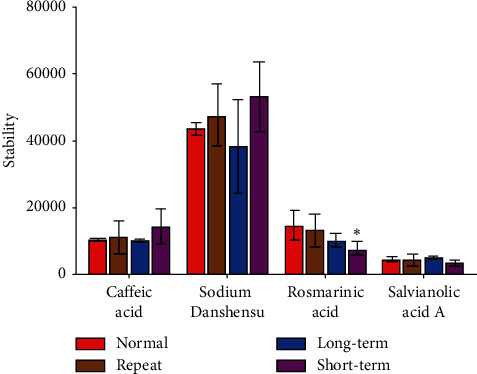
Comparison of the signal intensities for the four compounds under different storage conditions. Normal: normal experimental conditions; Repeat: after repeated rewarming for three times; Long-term: after long-term stored in a −80°C freezer; Short-term: after short-term placement at room temperature. ^*∗*^*P* < 0.05 vs. normal experimental conditions.

A sphericity test was performed before analysing the correlation between repeated data. There was no correlation between repeated data in this experiment (*P* > 0.05), and the measured data conformed to the Huynh–Feldt condition. Therefore, one-way analysis of variance was used for statistical analysis. There were no significant differences in signal intensities of DSS, CAA, and SAA under four conditions (*P* > 0.05). RA signal intensity showed lower level after preserving under room temperature for 24 h (*P* < 0.05, *P* = 0.047) compared with normal experimental conditions (Figure 4).

### 3.4. Distributions of the Four Salvianolic Acids

#### 3.4.1. Distribution of CAA in Different Tissues

**Figure 5:**
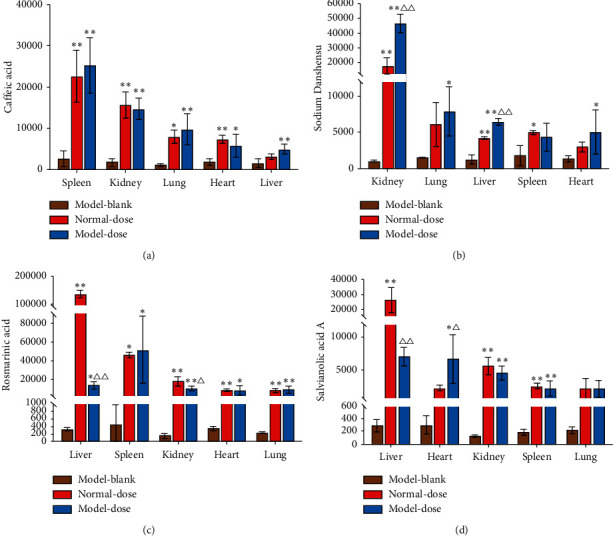
Signal intensities of four salvianolic acids in different tissues and groups. (a) Signal intensities of caffeic acid in different tissues and groups. (b) Signal intensities of Danshensu in different tissues and groups. (c) Signal intensities of rosmarinic acid in different tissues and groups. (d) Signal intensities of salvianolic acid A in different tissues and groups. Model-blank: model-blank group; Normal-dose: normal-dose group; Model-dose: model-dose group. ^*∗*^*P* < 0.05, ^*∗∗*^*P* < 0.01 vs. model-blank group. Δ*P* < 0.05, ΔΔ*P* < 0.01 vs. normal-dose group.

The CAA signal intensities in samples from the heart, spleen, kidney, lung, and liver were analysed. Both the model-dose group and normal-dose group showed significantly higher CAA signal intensities than the model-blank group in the heart, spleen, kidney, and lung (*P* < 0.05 or *P* < 0.01). Liver samples from the model-dose group had higher CAA signal intensities than those from the model-blank group (*P* < 0.01), but a significant difference was not observed between the normal-dose group and the model-blank group. There was no statistical difference in CAA signal intensities between the model-dose group and normal-dose group. Moreover, compared with other tissues, the spleen had the highest signal intensity of CAA (Figure 5(a)).

#### 3.4.2. Distribution of DSS in Different Tissues

Kidney, lung, liver, and heart samples from the model-dose group and kidney, liver, spleen samples from the normal-dose group showed higher DSS signal intensities than those from the model-blank group (*P* < 0.05 or *P* < 0.01). Compared with the normal-dose group, the signal intensities of DSS were significantly higher in the kidney and liver in the model-dose group (*P* < 0.01). Kidney had the highest DSS signal intensity compared with other tissues (Figure 5(b)).

#### 3.4.3. Distribution of RA in Different Tissues

Compared with the model-blank group, both the normal-dose group and model-dose group had higher RA signal intensities in the liver, spleen, kidney, heart, and lung (*P* < 0.05 or *P* < 0.01). Compared with the normal-dose group, liver and kidney samples showed lower signal intensities of RA in the model-dose group (*P* < 0.05 or *P* < 0.01). Samples from the normal-dose group were observed that the liver had the highest RA signal intensity; however, it was not observed in the model-dose group (Figure 5(c)).

#### 3.4.4. Distribution of SAA in Different Tissues

Compared with the model-blank group, liver, kidney, and spleen samples from the normal-dose group and heart, kidney, and spleen samples from the model-dose group showed higher SAA signal intensities (*P* < 0.05 or *P* < 0.01). Significant differences were observed in liver and heart samples between the normal-dose group and model-dose group (*P* < 0.05 or *P* < 0.01). The model-dose group showed higher SAA signal intensities in the heart samples (*P* < 0.05) and lower intensities in liver samples than the normal-dose group (*P* < 0.01). Compared with other tissues, liver tissues had the highest SAA signal intensity (Figure 5(d)).

## 4. Discussion

The Danshen decoction used in the present study has been reported to commonly use in promoting blood circulation and removing blood stasis [11], with a simple composition as *Salvia miltiorrhiza* Bunge, *Amomum villosum* Lour, and *Santalum album* L. The main chemical components of *Salvia miltiorrhiza* Bunge include lipo- and water-soluble components. Most of the liposoluble components are conjugated quinones and ketones. The water-soluble components are mainly phenolic acids such as CAA, DSS, RA, and SAA [4, 5]. Pharmacological studies have suggested that there were small quantities of liposoluble components such as tanshinone II-A and cryptotanshinone in *Salvia miltiorrhiza* Bunge [10]. Therefore, we presumed that the liposoluble components of *Salvia miltiorrhiza* Bunge would have little correlation with its traditional therapeutic effects and that the water-soluble components would be the main contributors [12].

Studies have shown that DSS could greatly improve blood rheology, lower lipid levels, inhibit lipid peroxidation, and have antitumour and other pharmacological activities [13]. SAA has protective effects on acute kidney injury induced by ischemia-reperfusion injury [14] and can greatly attenuate monocrotaline-induced hypertrophic damage of the myocardium, parenchymal injury, and collagen deposition in the lungs [15]. RA reportedly has a therapeutic effect on type 2 diabetes and hyperlipidaemia [16]. Studies have shown that CAA ameliorates cardiac damage in isoproterenol-induced myocardial infarction by maintaining lipid peroxide metabolism because of its free radical scavenging and antioxidant effects [17].

In the present study, after four weeks of administration of Danshen decoction, EF and FS were improved obviously in the model-dose group, which confirmed that Danshen decoction could improve the ischemic condition in a chronic myocardial ischemia model.

In addition, an HPLC-MS/MS method had been established in our previous study to determine the concentration of sodium Danshensu, protocatechualdehyde, caffeic acid, rosmarinic acid, and salvianolic acid A in rat plasma [18]. The results showed that the contents of the five compounds in blood were stable and were sufficient to detect. However, in the preliminary experiment of LESA-MS/MS, the signal intensity of protocatechualdehyde in the tissue was extremely low, so it was not included.

LESA-MS/MS was applied to analyse the distributions of four salvianolic acids. A stability investigation showed that DSS, CAA, and SAA were stable under four experimental conditions, but RA had a poor stability when placed at room temperature for 24 h. However, the samples were usually tested within 2 h of removal from the −80°C freezer. This short time out of the freezer might have little effect on the samples, and the results would be reliable for the distribution study.

Subsequently, distribution analysis showed that the signal intensities of DSS in the liver and kidney and SAA in the heart were higher in the model-dose group than in the normal-dose group. The reason for this might be that DSS has strong water solubility, and the liver and kidney are the main metabolic organs for DSS. It is reported that there is obvious inflammatory cell infiltration and an abnormal increase of vascular permeability in the ischemic zone of the myocardium [19]. The signal intensities of SAA in other organs decreased but increased particularly in the heart, indicating that myocardial ischemia would promote the transfer of SAA from other positions to the heart. The contents of the two salvianolic acids in the targets increased, which is basically consistent with the statement in Ben Cao Zheng that “Danshen is the medicine of heart, spleen, liver, and kidney” and is another proof of the effectiveness of Danshen decoction. These results have been confirmed in other studies. Zhang et al. [20] have found that DSS may have hepatic-protective effects on iron overload mice, and the experiment conducted by Gao et al. [21] suggested that DSS treatment in diabetic mice could help improve the renal clearance. It is reported that SAA protects the myocardium in canine experimental myocardial infarction models. Both oral and intravenous administration of SAA reduced the myocardial infarct area significantly [22]. A meta-analysis including 14 research studies demonstrated that salvianolic acids could exert cardioprotection through promoting angiogenesis in animal models of myocardial infarction [23].

Recently, several studies have analysed salvianolic acids using diverse analytical methods acids including liquid chromatography with ultraviolet detection (LC-UV), online solid-phase extraction coupled in series to liquid chromatography-tandem mass spectrometry (SPE-LC-MS), high-performance liquid chromatography-diode array detection (HPLC-DAD), HPLC-MS [24], high-speed countercurrent chromatography (HSCCC), and ESI-MS [25]. Although these methods are mature, pretreatment of the samples is always required, which leads to the inevitable loss of spatial information during the homogenization process [26]. LESA-MS/MS is a surface sampling technique that combines extraction of liquid from the tissue surface with nano-ESI-MS which can be used for spatial analysis of drug distributions and has been used historically to describe discrete points on the surfaces of tissue slices [27]. To date, no research has been conducted on salvianolic acids using nano-ESI-MS. Compared with other imaging-capable methods such as quantitative whole-body autoradiography and matrix-assisted laser desorption/ionization-MSI, LESA-MS/MS not only provides spatial distribution information for tissues but also greatly simplifies the pretreatment procedures and shortens the analysis time [26].

As a fully automated, chip-based multichannel MS method, LESA-MS/MS combines microliquid extraction from a solid surface and nano-ESI analysis to obtain information from tissue sections of interest [28]. Studies have shown that the quantitative and spatial distributions of exogenous chloroquine (CHQ) and CHQ metabolites in tissue slices can be rapidly and accurately analysed by LESA-MS/MS without extensive sample preparation such as tissue homogenization or HPLC separation. The results from LESA-MS/MS correlated well with those obtained by LC-MS [29].

## 5. Conclusions

Danshen decoction has the effect of improving the ischemic condition in a chronic myocardial ischemia model and is basically distributed in the heart, liver, and kidney, which is worth further clinical study. A LESA-MS/MS method was applied for the simultaneous determination of four salvianolic acids (CAA, DSS, RA, and SAA) in animal tissues. The method has characteristics in simple pretreatment of samples, sensitivity, and stability, which showed to be worthy of further application on drug distribution research studies.

## Data Availability

The data used to support the findings of this study are available from the corresponding author upon request.

## Abbreviations

TCM: Traditional Chinese medicine
LESA-MS/MS: Liquid extraction surface analysis coupled with tandem mass spectrometry
DSS: Danshensu
CAA: Caffeic acid
RA: Rosmarinic acid
SAA: Salvianolic acid A
EF: Ejection fraction
FS: Fractional shortening
HPLC: High-performance liquid chromatography
OCT: Optimal cutting temperature
MRM: Multiple reaction monitoring
LC-UV: Liquid chromatography with ultraviolet detection
SPE-LC-MS: Solid-phase extraction coupled in series to liquid chromatography-tandem mass spectrometry
HPLC-DAD: High-performance liquid chromatography-diode array detection
HSCCC: High-speed countercurrent chromatography
MSI: Mass spectrometry imaging
CHQ: Chloroquine
LC-MS: Liquid chromatography coupled with mass spectrometry.

## Supplementary Materials

Supplementary Materials Supplemental Figure 1: the linear graph of salvianolic acids. Supplemental Figure 2: HPLC chromatogram of caffeic acid. Supplemental Figure 3: HPLC chromatogram of rosmarinic acid. Supplemental Figure 4: HPLC chromatogram of salvianolic acid A. Supplemental Figure 5: HPLC chromatogram of Danshensu. Supplemental Figure 6: coronary angiography results four weeks after surgery. Supplemental Figure 7: echocardiography results at different time points. (A) Before left anterior descending ligation. (B) Four weeks after left anterior descending ligation, before administration. (C) Eight weeks after left anterior descending ligation, four weeks after administration.
