# Supplementary material for: Distribution Analysis of Salvianolic Acids in Myocardial Ischemic Pig Tissues by Automated Liquid Extraction Surface Analysis Coupled with Tandem Mass Spectrometry
Source: Evid Based Complement Alternat Med. 2020 Sep 14;2020:8476794. doi: 10.1155/2020/8476794 (PMC7509547; doi:10.1155/2020/8476794)
Supplement: Supplementary Materials — Supplemental Figure 1: the linear graph of salvianolic acids. Supplemental Figure 2: HPLC chromatogram of caffeic acid. Supplemental Figure 3: HPLC chromatogram of rosmarinic acid. Supplemental Figure 4: HPLC chromatogram of salvianolic acid A. Supplemental Figure 5: HPLC chromatogram of Danshensu. Supplemental Figure 6: coronary angiography results four weeks after surgery. Supplemental Figure 7: echocardiography results at different time points. (A) Before left anterior descending ligation. (B) Four weeks after left anterior descending ligation, before administration. (C) Eight weeks after left anterior descending ligation, four weeks after administration. [file 8476794.f1.docx]

## Supplementary Materials


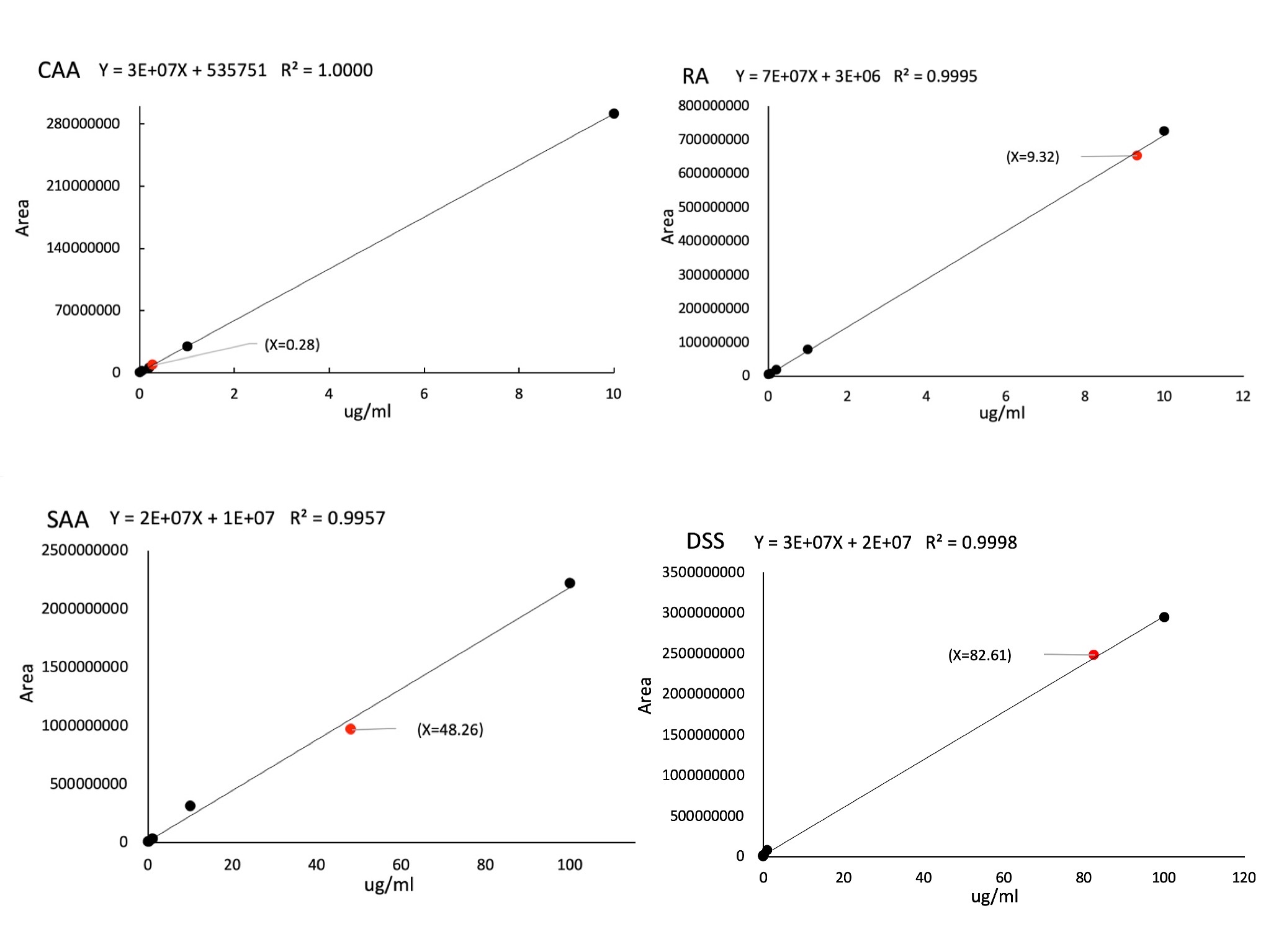


SUPPLEMENTAL FIGURE 1. The linear graph of salvianolic acids.


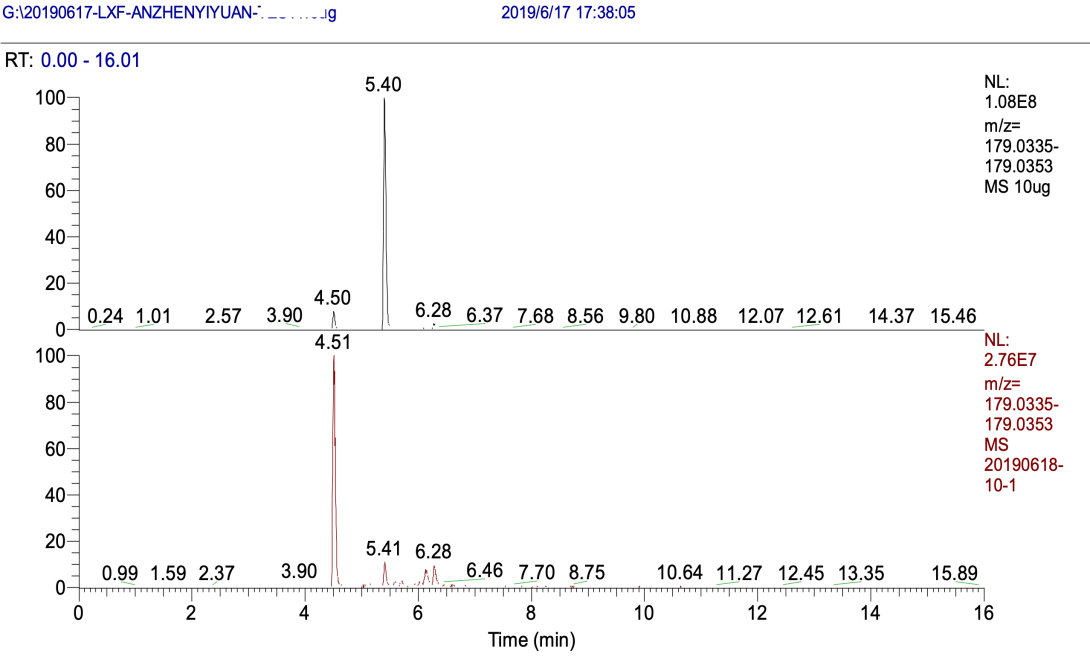
SUPPLEMENTAL FIGURE 2. HPLC chromatogram of caffeic acid.


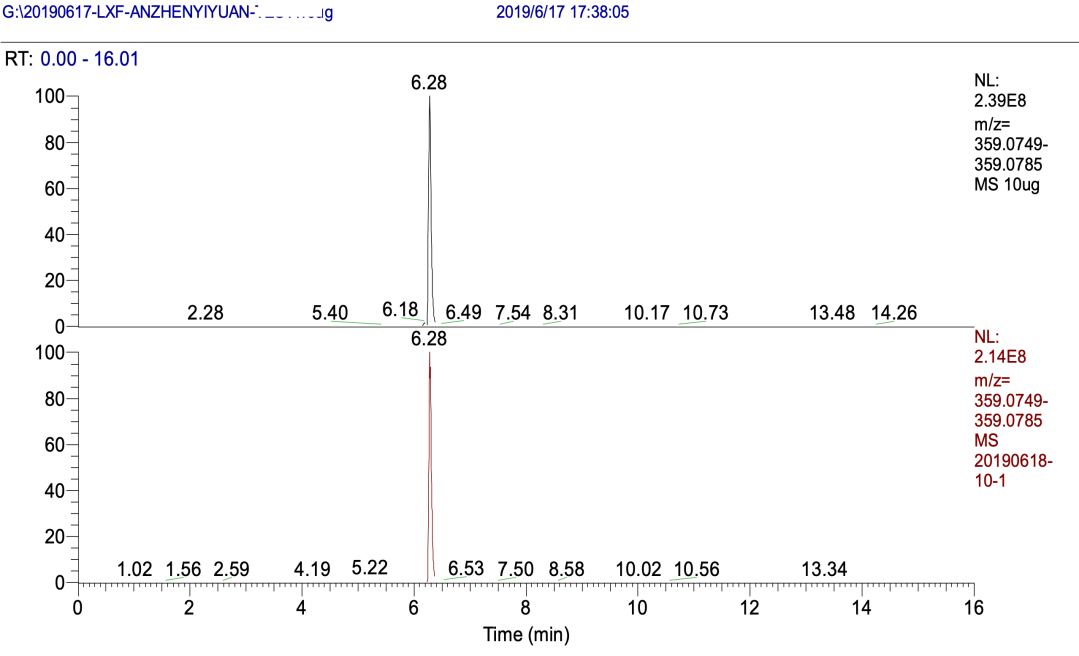
SUPPLEMENTAL FIGURE 3. HPLC chromatogram of rosmarinic acid.


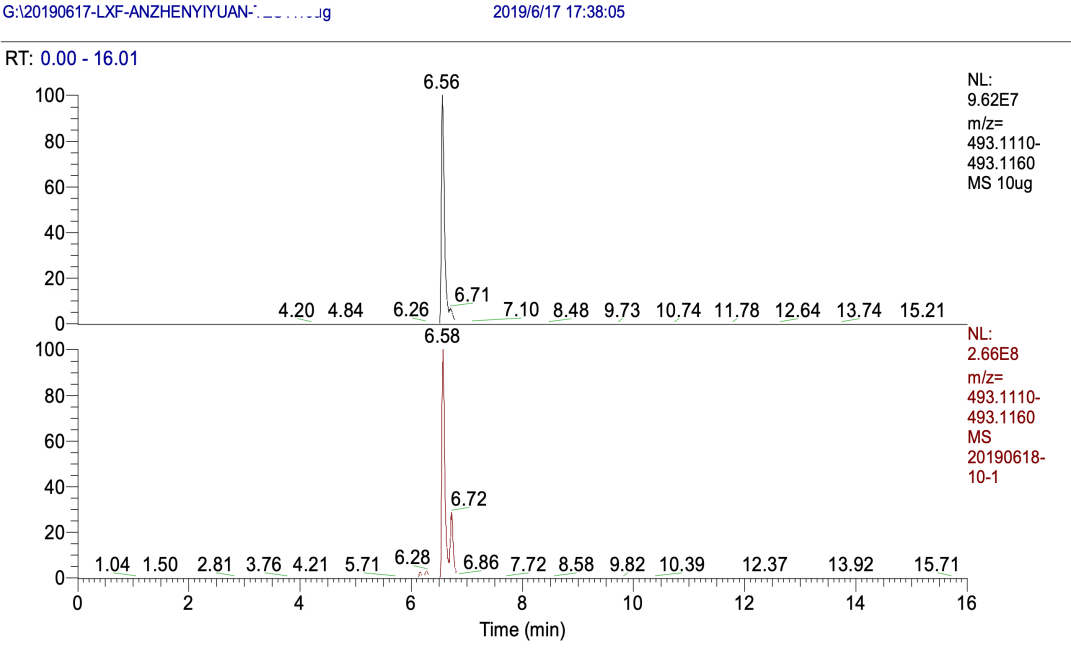
SUPPLEMENTAL FIGURE 4. HPLC chromatogram of salvianolic acid A.


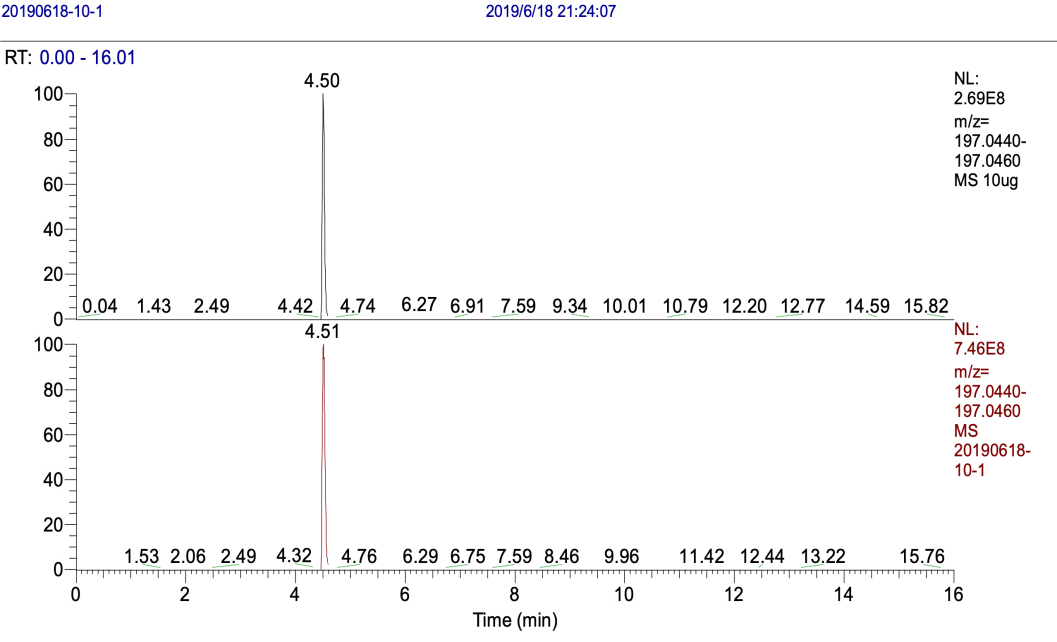


SUPPLEMENTAL FIGURE 5. HPLC chromatogram of Danshensu.


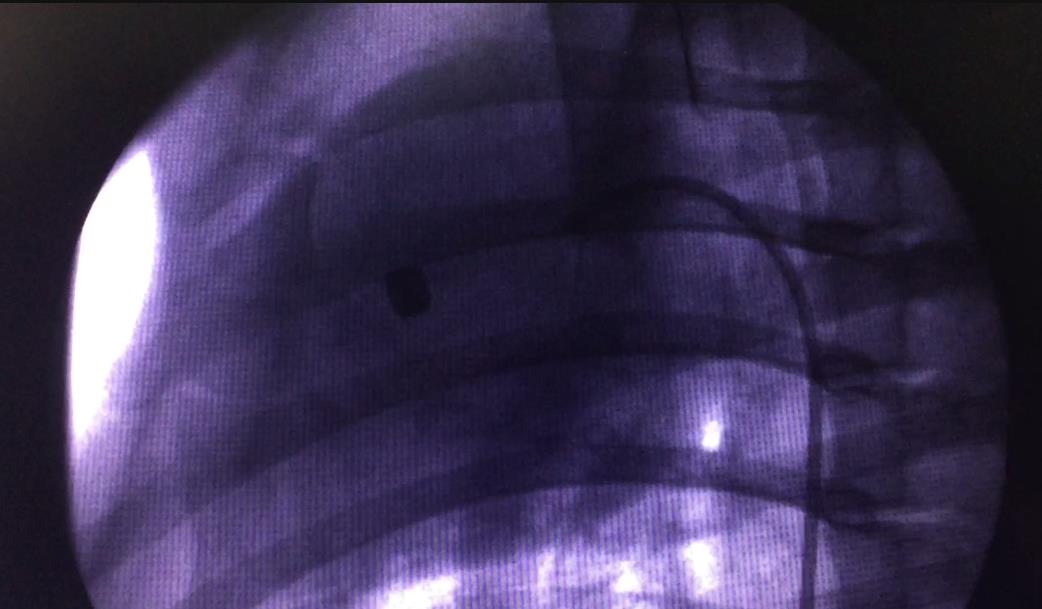


SUPPLEMENTAL FIGURE 6. Coronary angiography results four weeks after surgery.


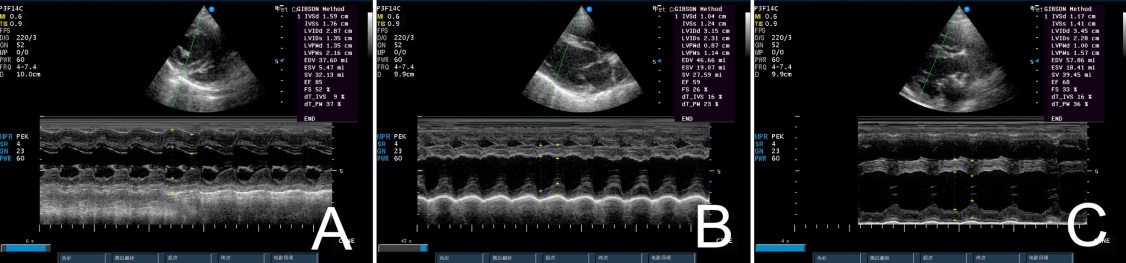


SUPPLEMENTAL FIGURE 7. Echocardiography results at different time points. (A) Before left anterior descending ligation. (B) Four weeks after left anterior descending ligation, before administration. (C) Eight weeks after left anterior descending ligation, four weeks after administration.
